# Current Advances in Lipid-Based Drug Delivery Systems as Nanocarriers for the Management of Female Genital Tuberculosis

**DOI:** 10.7759/cureus.74452

**Published:** 2024-11-25

**Authors:** Dhanashri D Chavan, Rohit R Bhosale, Vandana M Thorat, Akshay R Yadav, Sachinkumar V Patil, Bhagyesh U Janugade, Sarika J Patil

**Affiliations:** 1 Department of Pharmacology, Krishna Institute of Medical Sciences, Krishna Vishwa Vidyapeeth (Deemed to be University), Karad, IND; 2 Department of Pharmaceutics, Krishna Foundation’s Jaywant Institute of Pharmacy, Wathar, IND; 3 Department of Pharmaceutical Chemistry, Krishna Charitable Trust’s Krishna College of Pharmacy, Karad, IND; 4 Department of Pharmaceutics, Dr. Ashok Gujar Institute of Pharmacy, Karad, IND

**Keywords:** diagnosis, epidemiology, female genital tuberculosis, lipid-based drug delivery systems, pathogenesis

## Abstract

Female genital tuberculosis (FGTB) arises from *Mycobacterium tuberculosis* infection and can rarely be caused by *Mycobacterium bovis* or atypical mycobacteria. FGTB usually arises from tuberculosis (TB) that affects the lungs or other organs. The infection can enter the vaginal tract directly from abdominal TB or by hematogenous or lymphatic pathways. Menstrual dysfunction and infertility as a result of genital organ damage result from FGTB, which affects women's fallopian tubes, uterine endometrium, and ovaries. Consequently, FGTB remains a major worldwide health risk, posing challenges in its treatment due to the limited effectiveness of existing drugs and the resilient nature of the TB pathogen. Moreover, currently available antimicrobial drugs for FGTB suffer from inadequate bioavailability. Long treatment regimens are necessary because high doses often result in patient noncompliance and the emergence of drug-resistant strains of TB. Therefore, to improve TB therapy generally, especially FGTB, novel drug delivery techniques are essential. Because targeted drug delivery systems have the benefit of delivering higher drug concentrations directly to the infection site, fewer side effects have been reported. As a result, various lipid-based drug delivery systems as nanocarriers have been identified as successful antimicrobial drug delivery options, indicating their potential for treating FGTB.

## Introduction and background

Globally, tuberculosis (TB) causes 10 million instances of illness each year and 1.33 million deaths. It is still a major public health concern. Asia accounts for half of all TB-related deaths (85%), while Africa accounts for 30%. The disease is most common in people between the ages of 15 and 45 years. The rise of multi- and extensively drug-resistant (MDR and XDR) TB strains has aroused serious concerns due to their higher rates of morbidity and death [1]. In 1993, the World Health Organization (WHO) declared TB a global emergency in response to this global crisis, and they advised the directly observed treatment short-course (DOTS) technique, especially for developing nations [2]. For instance, by the end of 2005, the DOTS strategy had been successfully applied nationwide by the Revised National TB Control Programme (RNTCP) in India, leading to the diagnosis of about 71% of cases and a cure rate of more than 87%, which in turn produced a noteworthy seven-fold reduction in mortality [3]. Female genital tuberculosis (FGTB) is a kind of extrapulmonary tuberculosis (EPTB). Morgagni first described it in 1744 while conducting an autopsy on a young woman who had died of TB peritonitis, and the incidence of FGTB is rising among young women globally, highlighting the importance of addressing this specific manifestation of TB [4-7]. The purpose of this review is to report the recent findings related to lipid-based drug delivery systems as nanocarriers or nanomedicines for managing FGTB, and also to provide the recent findings or concise literature to the scientific community as well as researchers working on designing and developing lipid-based drug delivery systems to treat TB or EPTB, including FGTB. The strategy used for reporting these recent findings was to refer to the recent scientific literature through different scientific databases such as PubMed and ScienceDirect by using keywords including epidemiology, pathogenesis, and diagnosis of female genital tuberculosis, and also by using keywords like lipid-based drug delivery systems, nanocarriers, and nanomedicines for tuberculosis as well as female genital tuberculosis.

## Review

FGTB is responsible for causing menstrual dysfunction and infertility in women, and hence, timely identification of the condition and the administration of appropriate treatment regimens comprising well-balanced drug combinations and adequate dosages can effectively minimize organ damage and mitigate the risk of future infertility in affected women. Genital TB (GTB) was found in 9% of cases with EPTB, which is accounted for by genitourinary tuberculosis (27.1%) [7-11]. The reported incidence rates in various nations and areas varied. For example, infertility clinic cases in the USA indicate FGTB in about 1% of cases, although a similar incidence is observed in Scandinavian nations [12,13]. In Pakistan, the incidence ranges from 4% to 8% whereas in South Africa, it ranges from 15% to 21.1% [14-16]. In different areas of India, the reported incidence varies between 1% and 19% [17,18]. Tertiary centers often report higher incidence rates (up to 26%) due to the referral of complex cases [19], and women seeking assisted reproduction may have an incidence as high as 48% [20]. HIV infection impairs the immune system, which has led to a rise in the incidence of FGTB and EPTB in Africa and India. In developed nations, FGTB typically manifests at around 40 years of age. However, in Asia, where early marriage and childbearing are prevalent, the disease presents in a younger age group typically between 20 and 30 years old [8-11].

FGTB can lead to both primary and secondary infertility, affecting approximately 40-80% of FGTB cases, and infertility in the case of FGTB can result from various factors such as tubal factors, defective ovarian function, and uterine (endometrial) factors. Among the tubal factors are unilateral or bilateral hydrosalpinx (fluid build-up in the fallopian tubes) with or without obstruction, adhesions and tubo-ovarian mass formation from perisalpingitis, decreased tubal function due to FGTB-caused ciliary damage, and unilateral or bilateral fallopian tube blockage. These tubal abnormalities can adversely impact fertilization and the implantation of embryos thereby contributing to fertility issues in affected individuals. FGTB can lead to various endocrine dysfunctions, chronic anovulation (lack of regular ovulation), and even impact *in vitro* fertilization (IVF) cycles due to the antigonadotropic effect of *Mycobacterium tuberculosis*. Additionally, FGTB can lead to low progesterone production, which can result in luteal phase defects and poor embryo quality, which may be brought on by intrinsic oocyte factors. These factors, when combined, can lead to implantation failure, reduced pregnancy rates, and greater risks of loss in persons afflicted by FGTB [12,21]. Endometrial atrophy, or the thinned endometrial lining, and the development of synechiae, or adhesions inside the endometrium, can be the consequences of FGTB's substantial impact on endometrial receptivity, which also disrupts endometrial indicators and impairs vascularization. There have been reports of faulty or unsuccessful implantation in FGTB cases. FGTB induces a T-helper-1 cell (Th-1) immune response in lieu of the necessary Th-2 cell response, which obstructs successful implantation. One of the factors contributing to implantation failure in FGTB patients is the endometrium's transition from a Th-2 to a Th-1 response. Notably, latent FGTB has been identified as a significant cause of repeated IVF failure, predominantly observed in the Indian population, as it negatively impacts implantation [13,22].

Pathogenesis

**Figure 1:**
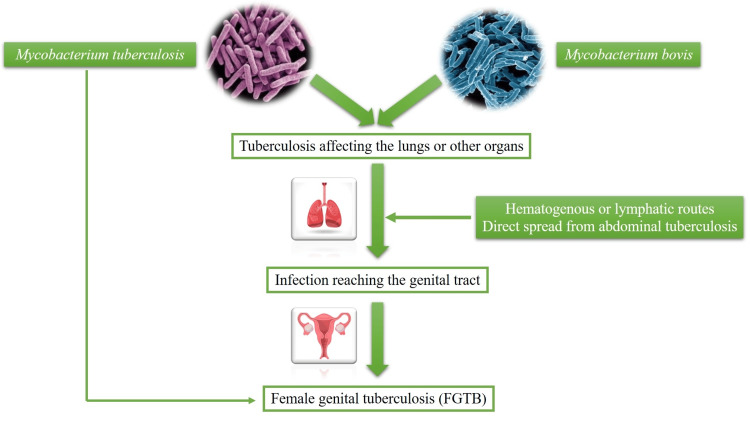
Pathogenesis of female genital tuberculosis (FGTB).

*Mycobacterium tuberculosis* is the main cause of FGTB, though it can also occasionally be brought on by atypical mycobacteria or *Mycobacterium bovis*. A person's susceptibility to TB is increased by a number of high-risk factors, including HIV infection, diabetes, renal illness, poverty, overcrowding, poor ventilation, and illegal drug use [8-12]. The majority of the time, hematogenous or lymphatic transmission is used to transmit GTB, which usually results from a primary TB infection in the lungs or other organs [21,22]. In rare instances, the virus may go straight from nearby organs like lymph nodes or the colon. Precautionary precautions during sexual activity can help lower the risk of contracting GTB infection. Additionally, contaminated semen may transmit GTB to a sexual partner in cases of active genitourinary TB in the male partner [12]. Given that it has been shown to be up to 80% effective in preventing severe forms of TB, the bacillus Calmette-Guérin (BCG) vaccine is routinely administered as a preventive measure in areas with a high TB incidence. Its preventive effectiveness, however, can differ greatly between members of the same group. The FGTB pathophysiology is shown in Figure 1.

Diagnosis

**Figure 2:**
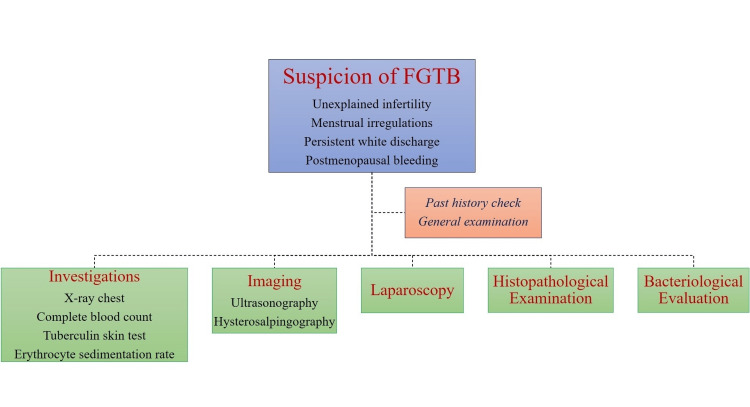
Diagnostic approaches for female genital tuberculosis (FGTB).

Moreover, FGTB can also result in vesicovaginal and rectovaginal fistulas, although they are rare [23]. The diagnosis of GTB, in particular FGTB, is still difficult to make despite the availability of a variety of diagnostic techniques. For this reason, identifying FGTB requires a thorough investigation, a high degree of suspicion, and a thorough and systematic clinical examination [24]. When treating patients with chronic pelvic inflammatory disease (PID) who do not respond to standard antibiotic treatment, unexplained infertility, irregular menstrual cycles, postmenopausal bleeding, or persistent vaginal discharge, doctors should consider the possibility of FGTB after ruling out genital neoplasias [25]. A number of risk factors should also be considered, including living in or recently visiting endemic areas, having a low socioeconomic background, abusing drugs, having a history of TB infection, being in close proximity to a patient with smear-positive pulmonary TB (PTB), and having HIV [26]. Establishing a diagnosis of FGTB requires a high index of clinical suspicion, a thorough history-taking, a thorough physical examination, a battery of tests to detect *Mycobacterium tuberculosis*, and imaging modalities to identify characteristic structural changes because there is currently no one diagnostic test that can conclusively confirm the diagnosis of FGTB [27]. Diverse diagnostic approaches for FGTB include general examination, hysterosalpingography (HSG), ultrasonography (USG), laparoscopy, histopathological examination (HPE), bacteriological evaluation, and culture methods. Figure 2 represents various diagnostic approaches for FGTB.

Lipid-based drug delivery systems as nanocarriers for managing PTB/EPTB/FGTB

Some of the goals of enhancing TB treatment generally include limiting the occurrence of multi-drug resistant TB (MDR-TB), reducing the period of therapy to enhance compliance, and easing the burden on public health infrastructure. Furthermore, the goal is to create TB medications that are safe, well-tolerated, and effective against drug-resistant strains of the disease. It is also critical to create TB medications that do not stimulate or inhibit liver cytochrome P450 enzymes to prevent drug interactions, especially when used in conjunction with antiretroviral therapy (ART), and to make treatment easier for patients who also have HIV. The World Health Organization (WHO) and the Centers for Disease Control and Prevention/Infectious Diseases Society of America recommend treating drug-sensitive FGTB for six months in the same manner as pulmonary tuberculosis (PTB) [28], despite the paucity of official research determining the optimal course of treatment for FGTB. It has been used to treat FGTB with good results and low rates of illness recurrence. The typical protocol entails two months of intensive treatment using isoniazid, rifampin, ethambutol, and pyrazinamide, followed by four months of continuing treatment using isoniazid and rifampin [29,30]. When treating MDR-FGTB patients, second-line medications are used for 24 months, divided into an 18-month continuation phase and a six-month intensive phase [31]. Even after receiving therapy, women with FGTB typically have a poor prognosis for fertility since the condition frequently results in irreparable damage to the genital organs. Post-treatment conception rates are estimated to range from 12% to 23% [32-34]. Though decisions for medication should not be solely based on polymerase chain reaction (PCR) results, some data suggest a positive PCR in the absence of clinical signs may indicate subclinical disease. In these situations, starting medication early can help avoid significant genital organ damage and possibly avert irreversible infertility [35].

Lipid nanoparticles, a state-of-the-art in drug delivery, have enormous potential for the prevention and treatment of infectious disorders; however, treating FGTB poses considerable obstacles. By utilizing these carriers, drugs can be made more soluble and bioavailable, potentially reducing their toxicity. It is possible to create sustained drug delivery systems that retain medications in the bloodstream for prolonged periods of time by using lipid-based nanoparticles. As a result, this method significantly lowers dosage frequency and improves patient compliance [36]. Furthermore, individuals with FGTB should be treated similarly to those with PTB, following a standard six-month course of treatment recommended by the World Health Organization (WHO), the Centers for Disease Control and Prevention, and the Infectious Diseases Society of America [28,37]. This has led to several recent reports of advancements in the field of lipid-based drug delivery systems as nanocarriers, some of which are related to FGTB therapy.

Liposomes

Liposomes have garnered a lot of interest as lipid-based nanocarriers for the transport of various medical components, such as therapeutic and diagnostic agents. These spherical vesicles are stabilized both *in vitro* and *in vivo* by the addition of cholesterol to phospholipids, which can be synthetic or natural molecules having both hydrophilic and hydrophobic qualities. Phospholipids spontaneously form a bilayer structure when dissolved in water, with the hydrophilic heads facing the aqueous phase and the hydrophobic tails pointing in the direction of one another [38], and this configuration enables the encapsulation of hydrophobic molecules within the lipid bilayer and the integration of hydrophilic molecules within the liposome's inner watery core. Morphologically, liposomes can be categorized into a number of different types, including giant unilamellar vesicles (GUVs), oligolamellar vesicles (OGVs), small unilamellar vesicles (SUVs), and large unilamellar vesicles (LUVs) [39,40]. A single lipid bilayer makes up unilamellar vesicles, which are further categorized as tiny if their size is less than 100 nm. On the other hand, enormous and large vesicles have sizes between ≥1 µm and >100 nm, respectively. Microscopy techniques can be used to discriminate between OGVs, which consist of two to five concentric lamellae, and multilamellar vesicles, which contain more than five lamellae. Medicines that are hydrophilic can be encapsulated in unilamellar vesicles with a big aqueous core, while lipophilic medicines are better suited for multilamellar vesicles [41]. Drug delivery using liposomes has been shown to be both safe and effective for a variety of illnesses, including FGTB. By allowing for targeted distribution, they improve the therapeutic efficacy of anti-TB medications, especially when given to the lungs. However, conventional liposomes have problems like their integrity being disrupted in the blood and gastrointestinal environments, which causes the medicine to be released quickly from the capsule. Furthermore, the reticuloendothelial system (RES) of macrophages rapidly removes them from the bloodstream. Hydrophilic polymers, including polyethylene glycol (PEG), are now being used increasingly commonly to coat liposomes in a hydrophilic layer that shields them from macrophage clearance to circumvent these limitations [42].

Many studies have been conducted on liposomes as potential drug carriers for anti-TB drugs. It has been discovered that liposomes are extremely appropriate and safe for the pulmonary delivery of several antimicrobial drugs, leading to improved treatment outcomes. Their biocompatibility and similarity to lung surfactants because of their phospholipid content is one of their main advantages. This resemblance reduces the possibility of local irritation [43,44]. Since liposomes are quickly absorbed by macrophages, they are well-known as proven drug carriers [45] and have garnered a lot of interest for their potential to target intramacrophage infections, such as *M. tuberculosis* [46], where the bacteria often reside [47]. Additionally, studies have concentrated on using liposomal encapsulation to enhance the effectiveness and pharmacokinetic characteristics of anti-TB medications. For example, the thin film hydration method and freeze-drying were used to make inhalable liposomal particles, which were then loaded with rifampicin. An enhanced composition including a drug-to-lecithin ratio of 1:5 and a mass ratio of 3:2 for soy lecithin and cholesterol showed a 79.25% encapsulation efficiency. When given by oral administration and intratracheal instillation in rat models, these liposomes exhibited improved pharmacokinetic patterns compared to the free drug and provided regulated and sustained drug release [48].

pH-sensitive liposomes have been developed as a method of inhibiting cargo digestion to get around this. These liposomes are made to release the medicine within when macrophages suck them up in early phagosomes before they even get to the phagolysosomes. This prevents deactivation and guarantees the intended biological action of the medication by allowing it to diffuse into the cytosol early. Premature "off-target" release of anti-TB medications is reduced and improved intracellular delivery to macrophages is provided by pH-triggered liposome release [49,50].

Although liposomes appear to be a dependable delivery system for anti-TB medications, their expensive cost prevents broad use. The employment of expensive ingredients and sophisticated equipment, including microfluidic systems, is the primary cause of liposome formulation costs [51], especially when creating pH-sensitive liposomes, which frequently call for specific lipids like DOPE (dioleoylphosphatidylethanolamine) and CHEMS (cholesteryl hemisuccinate). To overcome this obstacle, scientists looked into the economical and practical encapsulation of anti-TB medications using crude soybean lecithin, a naturally occurring lipid combination [52,53]. Early studies revealed that in terms of isoniazid (INH) liposomal encapsulation, crude soybean lecithin worked better than purified soybean lecithin. Release tests at pH 7.4 also demonstrated a sudden release pattern, suggesting the likelihood of an early burst release and "off-target" loss of INH before reaching the infected area. Therefore, the present focus of research is on improving the transport and liposomal encapsulation of INH, a small hydrophilic drug. Further study was done on the liposomal encapsulation of isonicotinic acid (4-hydroxy-benzylidene)-hydrazide (INH-HB), an isoniazid hydrazone derivative that allows for pH-dependent controlled release, to address the issue of liposome leakage from crude soybean lecithin liposomes [54]. The hydrazone derivative was successfully liposome-encapsulated utilizing crude soybean lecithin for cost-effective development. The INH-HB-loaded liposomes showed significant pH-responsive drug release; they reached 100% at pH 4.4 compared to 22% at neutral pH (7.4). Based on this information, Nkanga and Krause [55] conducted a thorough study to develop a versatile liposomal tool for anti-TB theranostic applications by conjugating INH to zinc phthalocyanine, a hydrophobic fluorescent tag, utilizing hydrazone linkages (mixing fluorescence bioimaging with drug delivery). The artificial dual liposomes demonstrated significant penetration into human lung fibroblast and epithelial cell biological membranes in addition to pH-dependent, regulated co-delivery of two potent anti-TB drugs with good biocompatibility. However, because of this liposomal system's intricacy, the total drug dose is called into doubt [56].

Niosomes

While niosomes resemble liposomes in shape, their composition is distinct. Unlike ionic surfactants, non-ionic surfactants generate a bilayer structure that is the basis for niosomes. These bilayers consist of mixtures of non-ionic surfactants and hydrated cholesterol. Surfactants for niosomes include polysorbates (like Tweens®), sorbitan fatty acid esters (like Spans®), and polyethoxy fatty ethers (like Brijs®) [57]. Like liposomes, the vesicle's bilayer is made stiffer and more stable by the addition of cholesterol. It has been discovered that the addition of non-ionic surfactants causes the vesicles to enlarge, hence increasing their encapsulation effectiveness [58]. Drugs that are hydrophilic can be added to the aqueous core of niosomes, but substances that are lipophilic are enclosed in lipophilic bilayers. Comparable in function to liposomes, niosomes can improve the solubility and bioavailability of medications. But compared to traditional liposomes, niosomes have better chemical stability, are less expensive, and have a greater penetrating capacity [59,60]. Moreover, niosomes have a higher capacity for penetration than traditional liposomes [61-63].

Lipid-based structures called niosomes have been thoroughly investigated as nanocarriers to improve the pharmacokinetics and regulate the biodistribution of anti-TB medications. In one investigation, rifampicin-loaded niosomes were investigated using Span® 85 and cholesterol in different molar ratios [62,64]. Rats were used in *in vivo* distribution tests, and the results indicated that after the drug was administered via the caudal vein, about 65% of the encapsulated rifampicin was found in the lungs. Another study examined niosomal delivery systems loaded with isoniazid by means of controlled release evaluations and biodistribution analyses. Reverse phase evaporation was used to generate niosomes, which showed prolonged and sustained drug release characteristics over a 30-hour period. Furthermore, these drug-loaded niosomes demonstrated strong cell penetration (about 62%) on macrophages, an excellent characteristic for addressing mycobacteria [62,65].

Studies on niosome technology for TB also investigated the influence of surface properties on niosomal particle activity. It was studied how pyrazinamide loaded into niosomes made of cholesterol and Span® 60 or 85, together with the inclusion of dicetyl phosphate (DCP) or stearyl amine to impart positive or negative surface charges [66]. The formulation with the maximum entrapment was the one containing cholesterol and Span® 60 at a molar ratio of 4:2. The best encapsulation effectiveness was shown by negatively charged niosomes, which were followed by neutral niosomes. *In vivo* biological studies on guinea pigs infected with *M. tuberculosis* showed that pyrazinamide was more effective when it was encapsulated in niosomes.

Another study looked into the efficacious co-encapsulation of anti-TB drugs in niosomes [67]. Rifampicin, isoniazid, and pyrazinamide, three first-line antitubercular drugs, were encapsulated in exceptionally stable niosomes made of Triton X 100, PEG 2000, water, and Span® 80. Every medication demonstrated strong system compatibility and stability in addition to great encapsulation efficiency. The biocompatible surfactant tyloxapol was also used to encapsulate rifampicin, isoniazid, and pyrazinamide in niosomes [68]. The investigation showed that each medication had a high encapsulation efficiency, and the final formulations had good stability. According to localization studies, pyrazinamide mostly adsorbed on the surface of head groups, whereas isoniazid and rifampicin most likely lived in the film bilayer. Isoniazid and pyrazinamide showed sustained release characteristics, although rifampicin released more quickly because of its different solubility. To be in line with existing anti-TB therapy regimens, the findings called for additional clarification of the *in vivo* activity of these multidrug-loaded niosomes. To sum up, niosomes have demonstrated promise as anti-TB medication nanocarriers, providing enhanced biodistribution and regulated release. To evaluate their antitubercular effectiveness, maximize encapsulation efficiencies, and create multidrug-loaded formulations that complement existing treatment plans, more study is necessary.

Solid Lipid Nanoparticles

Solid lipid nanoparticles (SLNPs), a type of colloidal carrier, are composed of biodegradable lipids that maintain their solid state at both ambient temperature and body temperature [69]. Long-chain triglycerides or partial glycerides, phospholipids, fatty acids, and waxes are among the lipid components used to create SLNPs [70]. It is customary to include a stabilizer in the form of a surfactant, such as lecithin, poloxamer 188, or polysorbate 80, at concentrations between 1% and 5% (w/v) to guarantee stability [71]. The solid lipid matrix can be used to encapsulate bioactive substances, especially lipophilic molecules, which can then be released under regulated conditions. The majority of SLNPs are spherical in shape and range in particle size from 10 to 1000 nm [72,73]. Their safety and effectiveness are increased by the formulation's inclusion of physiological lipids. SLNPs overcome the shortcomings of conventional lipid and polymeric nanoparticles while providing the benefits of both. SLNPs show promise in enhancing medication stability and bioavailability via several modes of delivery.

The creation of SLNPs as carriers for improving the solubility, bioavailability, and effectiveness of anti-TB medications has attracted a lot of study attention in recent years. These investigations cover a range of phases, from sophisticated evaluations of pharmacokinetics and antimycobacterial action to formulation design and optimization. For example, a study examined the effects of pre-freezing conditions on the deep lung deposition of SLNPs loaded with rifampicin, which were prepared using stearic acid and sodium taurocholate [74].

Another component of SLNP design involves optimizing their composition to add certain capabilities, such as ionic charges or receptor-specific ligands for targeted distribution. An example of the impact of surface charges is provided by the coating of chitosan applied to SLNPs loaded with rifampicin [75]. The coating was confirmed by Fourier-transform infrared spectroscopy (FTIR) and surface charge analysis. In alveolar epithelial cells, chitosan-coated SLNPs (C-SLNPs) showed increased permeability and mucoadhesive characteristics along with a positive surface charge.

However, research on macrophage absorption and antitubercular activity is required to evaluate how well C-SLNPs work as a treatment. In SLNP research, the controlled and prolonged release of encapsulated medications has also been studied. For example, the prolonged release of rifampin from SLNPs consisting of Tween® 80/poloxamer 188 and cetyl palmitate was noted, leading to improved antimycobacterial effectiveness [76]. The formulation's stability profile might be impacted by the less stable negative surface charges that were observed. Furthermore, while combination therapy is advised for TB, the therapeutic applicability of this monotherapy may be restricted.

A noteworthy study assessed the chemotherapeutic potential of nebulized SLNPs comprising pyrazinamide, isoniazid, and rifampicin in the context of multidrug therapy [77]. These stearic acid-based SLNPs showed effective drug encapsulation, which enhanced the drug's residence time and bioavailability in particular organs. In guinea pigs, nebulized drug-loaded SLNPs demonstrated strong anti-*Mycobacterium tuberculosis* activity without causing hepatotoxicity. However, further investigations are needed to understand the uptake of SLNPs by macrophages for effective treatment of latent TB. To sum up, studies on SLNPs for TB have concentrated on formulation design, development, and optimization in addition to evaluations of pharmacokinetics and antimycobacterial action. These investigations have looked at a number of topics, including surface charges, prolonged release, pre-freezing conditions, and multidrug therapy. They have also shed light on the possibility of using SLNPs as carriers for more effective TB treatment.

Nanostructured Lipid Carriers

Nanostructured lipid carriers (NLCs) have surfaced as a promising advancement in lipid nanoparticle technology following the first generation of lipid nanoparticles known as SLNPs [78,79]. NLCs are distinguished by an unstructured matrix architecture that includes an aqueous phase containing one or more surfactants and a core matrix made of a combination of liquid and solid lipids [80-82]. For the formulation, solid and liquid lipids are typically blended in varying ratios (70:30 to 99.9:0.1), with the surfactant level typically being between 1.5 and 5% (w/v) [80]. Within the solid matrix of NLCs, the integration of suitable and biodegradable liquid lipids provides multiple advantages over SLNPs with regard to drug delivery.

The effectiveness of NLCs as medication delivery systems for TB treatment has been shown in numerous research. Mannosylated NLC, for instance, was created to specifically deliver rifampicin to alveolar macrophages [83]. In comparison to a rifampicin solution, these NLCs showed an appropriate size (160 nm) for deep lung deposition and led to an increase in drug accumulation in the lungs. NLC loaded with mannosylated rifampicin showed increased absorption efficiency in cells and alveolar macrophages, which inhibited *M. avium*'s intracellular proliferation in macrophages [81].

Rifabutin-encapsulating mannosylated NLC was developed in a different work [84]. These NLCs showed a pH-dependent release profile, releasing more quickly at pH 5.0, indicating that they could be specifically delivered to the acidic phagolysosomes that house the agent that causes TB. Nevertheless, no pharmacokinetic or pharmacodynamic characteristics were given. Additionally studied are functionalized NLCs such as tuftsin-modified peptide-coated NLC [85]. Compared to free rifampicin, these targeted NLCs showed improved *in vitro* activity against *M. tuberculosis* and higher uptake by alveolar macrophages. These compositions' antimycobacterial properties, however, were not disclosed. Polymer-coated NLC has been investigated for anti-TB medication delivery; these include chitosan-acemannan conjugate-modified NLC and polysorbate-coated sophorolipid-based NLC [86,87].

These formulations showed promise for the treatment of TB due to their good cell internalization and prolonged drug release. However, these studies lacked evaluations of anti-TB efficacy. The capability of NLC technology to co-load various medicinal compounds with varying solubilities is a benefit. NLC loaded with isoniazid and rifampicin successfully co-localized with several cellular compartments in macrophages and accomplished phagolysosomal trafficking [88]. When compared to unformulated medicines, the pharmacokinetic profile and relative bioavailability of the dual-drug-loaded NLC were enhanced by oral administration. Moreover, NLC has demonstrated promise for oral drug delivery, especially for copper (II) complexes that have limited water solubility and strong antibacterial action but poor *in vivo* efficacy [89]. When compared to free copper (II) complexes, the encapsulation of copper (II) in NLC decreased acute toxicity and increased anti-TB activity [90,91]. Finally, NLC has shown great promise as a platform for managing TB, including targeted delivery, co-loading capabilities, enhanced pharmacokinetics, and the possibility of oral drug delivery. To progress their translational development as sophisticated nano-formulations, more study is necessary.

Emulsions

Colloidal dispersions consisting of two immiscible liquids are commonly referred to as nano- and microemulsions. These systems usually include an oil phase, an aqueous phase, a cosolvent or cosurfactant, and a surfactant [92,93]. According to the exterior phase, the hydrophobic and hydrophilic areas of the nanoparticles in these systems are arranged in a core-shell configuration [94]. Nano- and microemulsions include both water-in-oil and oil-in-water emulsions. They can be made using high-energy techniques like microfluidization or high-pressure homogenization, or they can be created spontaneously [95,96]. Although transparency, isotropy, and low viscosity are commonalities across nano- and microemulsions, their characteristics are different. Unlike nanoemulsions, which are typical emulsions with a particle size precisely below 200 nm, microemulsions are "swollen micelle" systems in which the internal phase is integrated into the surfactant micelle core under specific compositions and environmental conditions [97-99]. From a physiochemical standpoint, microemulsions are thermodynamically stable systems, whereas nanoemulsions are thermodynamically unstable systems [98]. While the individual components of microemulsions have a higher free energy than the microemulsion itself, nanoemulsions have a higher free energy than their individual components. Because of their slow mass transit and high-energy barriers, nanoemulsions are kinetically stable systems whose destabilization process is slowed down [100].

It has been established that nano- and microemulsions are the best vehicles for delivering drugs via a variety of delivery channels, such as parenteral, pulmonary, ophthalmic, and oral [101-103]. Unfortunately, problems with drug hydrolysis, long-term stability, and poor palatability have restricted their use, and to address these issues, self-(nano)emulsifying drug delivery systems, or S(N)EDDS, have been created. S(N)EDDS are isotropic mixtures of oils, surfactants, and cosurfactants or cosolvents that can spontaneously form (nano)emulsions with droplet sizes of 200 nm or less when exposed to gastrointestinal fluids [104]. Typically, these systems have high concentrations of surfactants and cosurfactants (over 60%) and oils (less than 30% w/w). Benefits of S(N)EDDS include increased patient compliance, palatability, and chemical and physical stability. They do not include water and are neatly packaged into capsules for a single dose. S(N)EDDS also has the advantage of greater drug loading, better drug absorption, controlled delivery, targeting, and less variability caused by dietary influences. They increase the bioavailability of drugs and can be easily manufactured and scaled up.

Therapeutic emulsions, especially microemulsions, have been extensively studied for the administration of anti-TB medications. In one investigation, oleic acid, Tween® 80, ethanol, and phosphate buffer were used to create a microemulsion that included rifampicin. Rifampicin caused structural changes in the microemulsion, converting it from a water-in-oil to an oil-in-water system that remained stable during infinite dilution. Dissolution studies show that the formulation has the potential to be further explored, as seen by the controlled release of rifampicin from the oil-in-water emulsion droplets [105]. Further studies concentrated on adding isoniazid, another anti-TB medication, to the same Tween® 80-based microemulsion. When rifampicin was dissolved, the emulsion structure underwent significant alterations, changing from a water-in-oil to an oil-in-water microemulsion. Remarkably, the oil-in-water microemulsion that was produced maintained its stability when hydrophilic isoniazid was added. The oil-in-water microemulsion included isoniazid molecules, according to spectroscopic analyses. Despite the fact that the microemulsion is a dual formulation containing both rifampicin and isoniazid, the study did not offer bioassay data to assess its efficacy for treating TB [106].

By co-loading extra first-line anti-TB medications in compliance with multidrug regimens, subsequent research attempts sought to increase the therapeutic potential of microemulsions. Microemulsions containing the different solubilities of pyrazinamide, isoniazid, and rifampicin were subjected to structural investigation. The microstructure of non-ionic Brij® 96 microemulsions and the solubilization sites of these antitubercular drugs were examined. It was found that rifampicin and isoniazid were located at the interface, with rifampicin being closer to the oil body and isoniazid being closer to the hydrophilic side. Conversely, pyrazinamide stayed in the dispersion medium. The position and stability of the medications within the microemulsions were verified by differential scanning calorimetry. Nevertheless, there was no antimycobacterial activity data available to assess the encapsulated medications' effectiveness as the best anti-TB therapy [107]. Co-encapsulation of isoniazid, pyrazinamide, and rifampicin in Brij® 96 microemulsions was investigated in a different study. It was discovered that the antitubercular medication solubilization sites in the co-loaded microemulsions and the single-drug-loaded microemulsions were identical. Isoniazid and pyrazinamide showed an abnormal release pattern in *in vitro* release tests, but rifampicin showed a diffusional Fickian release mechanism. The cytotoxicity of the formulation was evaluated by the concentration of the microemulsion and the colloidal structure. Nevertheless, no biological research was done to evaluate how the drug's antitubercular action and stability improved after being encapsulated in the microemulsions [108].

Additionally, a solid self-nanoemulsifying drug delivery system (SNEDDS) containing rifampicin was created using Capmul® MCM C8 as the oil, Labrasol®, and Cremophor-EL. At various pH levels, the solubilization of rifampicin with Labrasol® led to an instantaneous release of the medication. Improved intestinal permeability of rifampicin was shown by *in vivo* permeation and biopsy investigations, and a strong *in vivo-in-vitro* correlation was noted, with about 96% anticipated systemic absorption. The rifampicin-loaded solid SNEDDS had better pharmacokinetic properties than the rifampicin solution, indicating that it would be a good alternative for the treatment of TB. However, more research is required to address drug resistance issues and assess the potential efficacy of the new SNEDDS formulations by co-loading additional anti-TB medications and performing antimycobacterial bioassays [109]. In general, studies on therapeutic emulsions have yielded important information about the stability of their structure and the addition of anti-TB medications. To analyze their antimycobacterial activity and determine how effective they are in treating TB, more research is necessary.

Table 1 represents a few examples of lipid-based drug delivery systems designed to encapsulate and deliver anti-TB drugs.

**Table 1:** Lipid-based drug delivery systems designed to encapsulate and deliver anti-TB drugs. TB: tuberculosis.

Drug delivery system	Drug(s)	Composition	Outcome	Reference
Liposomes	Isoniazid Pyrazinamide	Phosphatidylcholine, cholesterol	Attempt for multiple drug encapsulation in liposomes; co-encapsulation of isoniazid and pyrazinamide was successful	[110]
	Rifampicin	Soy phosphatidylcholine/hydrogenated derivative, cholesterol, oleic acid	Good cellular uptake and less toxicity toward alveolar epithelium for the formulation without oleic acid	[111]
	Rifampetine	Hydrogenated soy phosphatidylcholine, cholesterol	Antimicrobial efficacy without cytotoxicity in A549 cells	[112]
	Zinc phthalocyanine	Dipalmitoylphosphatidylcholine, cholesterol	Inactivation of sensible and multidrug-resistant strains of *M. tuberculosis* by photodynamic activity	[113]
Niosomes	Rifampicin	Span^®^ 85, cholesterol	Good distribution with lung affinity of approximately 65% due to the controlled size of particles	[64]
	Pyrazinamide	Span^®^ 60/85, cholesterol, dicetyl phosphate/stearyl amine	Improved drug efficacy in guinea pigs infected with *M. tuberculosis*	[66]
	Isoniazid	Span^®^ 20/60, cholesterol, dicetyl phosphate	Prolonged delivery in treated sites and high macrophage uptake of negatively charged particles	[65]
Solid lipid nanoparticles (SLNPs)	Rifampin	Cetyl palmitate, Tween^®^ 80/poloxamer 188	Improved antitubercular activity and sustained release of rifampin	[76]
	Rifampicin	Cetyl palmitate, chitosan	Higher *in vitro* mucoadhesive properties and permeability in alveolar epithelial cells	[75]
	Ethambutol	Compritol, Tween^®^ 80	Biocompatible, non-toxic particles, dry powder inhaler suitable for pulmonary delivery	[114]
Nanostructured lipid carriers (NLCs)	Rifampicin	Polyoxyethylene 40 stearate, caprylic/capric triglyceride, polyoxyl 40 hydrogenated castor oil, poloxamer 407, cetyltrimethylammonium bromide	Improved uptake of drug in alveolar macrophages	[83]
	Rifampicin	Lipoid S-75, Tween 80, poloxamer 188, Precirol^®^ ATO-5, glyceryl distearate, squalene	Enhancement of pharmacokinetic parameters and improvement of drug bioavailability	[115]
Emulsions	Rifampicin	Oleic acid, phosphate buffer, Tween^®^ 80, ethanol	Controlled release of rifampicin achieved	[105]
	Isoniazid	Oleic acid, phosphate buffer, Tween^®^ 80, ethanol	Stable formulation	

Case studies and clinical findings

Advances in laboratory studies intended to improve the delivery of therapeutic agents for the treatment of female genital infections have led to the resolution of the laboratory barrier for many studies that are presently moving through various stages of clinical research. In one investigation, a comparison was made between a six-month and a nine-month course of anti-tuberculous therapy for patients with FGTB, wherein, the study involved a randomized controlled trial with 175 women who presented with infertility and were diagnosed with FGTB through clinical examinations and investigations. DOTS was used to administer a nine-month intermittent anti-tuberculous therapy to Group I, which included 86 women, and a six-month anti-tuberculous therapy to Group II, which included 89 women. The primary objectives (full cure, partial response, no response) and secondary endpoints (pregnancy rate, recurrence rate) were evaluated at several intervals during the course of the treatment, wherein all patients underwent a year of follow-up after therapy ended to monitor any disease recurrence and any subsequent pregnancies. Both the six- and nine-month groups' complete clinical response rates (95.3 vs. 97.7%) did not differ statistically significantly, according to the study; however, two patients in the six-month group and four patients in the nine-month group needed category II anti-tuberculous therapy because of disease recurrence. Notably, there was no difference in the incidence of full cure, recurrence, or pregnancy among the groups receiving either six months or nine months of intermittent directly monitored treatment short course anti-tuberculous therapy for FGTB [116].

Additionally, the ovarian reserve was assessed in infertile individuals with GTB who planned to undergo IVF in comparison to women who were found fertile to identify and evaluate the outcomes, wherein a cross-sectional study comprising 104 women with GTB diagnoses and an equal number of healthy controls was carried out at an outpatient gynecology facility. To assess the ovarian reserve in both cohorts, serum levels of follicle-stimulating hormone (FSH), luteinizing hormone (LH), estradiol, and inhibin B were evaluated on the third day of a normal menstrual cycle, and on the same day, estimates of ovarian stromal blood flow, ovarian volume, and the number of antral follicles were also made. The people with GTB exhibited significantly lower mean levels of inhibin B and significantly greater mean levels of FSH and LH compared to the controls; on the other hand, the average number of antral follicles and mean ovarian volume were significantly lower in the GTB group. This difference persisted for each ovary's mean peak systolic velocity and pulsatility index. The information obtained from the analyzed markers suggested that women with GTB had a reduced ovarian reserve [117].

In one of the studies, infertile women underwent a comparative analysis of GTB diagnostic techniques and an assessment of their reproductive results post antitubercular therapy (ATT). Among the diagnostic procedures used were endometrial aspiration (EA), peritoneal washing (PW) for histological assessment, PCR, acid-fast bacilli (AFB) smear, and *Mycobacterium tuberculosis* culture. Laparoscopy and hysteroscopy were also carried out, and a six-month course of ATT was administered to women who had positive laboratory results and/or definitive/probable GTB findings via laparoscopy. Among the 196 women who were recruited, 187 had a laparoscopy, and 118 of them (60.2%) had GTB diagnosed as a result. PW PCR was positive in 7.6% of cases, while EA PCR produced positive results in 41.3% of cases. Out of the 118 women who had treatment for GTB, 22.9% became pregnant naturally, with 74.1% demonstrating a positive EA PCR and 59.3% displaying positive laparoscopic results. The study highlighted the fact that no single test is able to identify every case of FGTB, and it recommended using a combination of tests to increase detection rates [118].

Additionally, research was carried out to determine the prevalence of a recently discovered entity known as "Sharma's sigmoid colonic adhesive band" in patients with FGTB during laparoscopy. It involved 148 infertile women who had received a diagnosis of FGTB based on microbiological or laparoscopic results within the previous 10 years wherein diagnosis of FGTB was confirmed by a number of techniques, including laparoscopic findings, positive gene Xpert, positive PCR, or histological proof of epithelioid granuloma, as well as endometrial or peritoneal biopsy microscopy or culture for AFB. Interestingly, 49 (33.10%) FGTB patients experienced the new laparoscopic sigmoid colonic adhesive band; this discovery, known as "Sharma's sigmoid colonic adhesive band," stands out as an important observation in FGTB patients [119].

Future directions

Novel strategies for healing the endometrium, ovaries, and damaged fallopian tubes caused by TB infection are being researched. These strategies include stem cell therapy, nanotechnology, and colostrum. Injectable regimens are important for treating TB of all kinds, including MDR and rifampicin-resistant TB. Recently, the WHO suggested a bedaquiline and delamanid-based injection-free regimen to treat drug-resistant forms of *Mycobacterium tuberculosis*. Research on developing new medications to attack FGTB is still ongoing wherein the strategy seeks to reduce therapy duration and enhance the outcomes. Furthermore, novel and innovative drug delivery systems such as nanocarriers or nanomedicines have come into the limelight, particularly those involving lipid-based carriers for managing TB as well as EPTB, including FGTB.

## Conclusions

Traditional drug delivery systems, which often require frequent high doses, can be a significant barrier to patient adherence, ultimately contributing to treatment failure and the rise of MDR-TB strains. To address these challenges, innovative delivery methods, particularly those involving lipid-based nanocarriers such as liposomes, niosomes, SLNPs, NLCs, and nano- or microemulsions have emerged. As per the recent literature and different research outcomes reported by different researchers, all these drug delivery systems have demonstrated great promise in enhancing the bioavailability, stability, and safety profiles of anti-TB medications. Moreover, a key advantage of lipid-based systems is their potential to improve targeted delivery, which is essential for the effective treatment of TB. However, while these systems hold considerable potential, their widespread use remains limited. Challenges such as scaling production, high costs for patients, and the need for specialized equipment prevent lipid-based nanomedicines from reaching the market in large numbers. Despite these hurdles, the potential of lipid-based drug delivery systems to revolutionize PTB or EPTB treatment is clear, thereby underscoring the need for continued research and development to make these technologies commercially viable and widely accessible.
